# Associations of Pretreatment Hemoglobin, Body Mass Index, and Leukocyte Count with Acute and Late Toxicity During and After Radiotherapy for Rectal Cancer

**DOI:** 10.3390/cancers18213475

**Published:** 2026-10-28

**Authors:** Nouf M. Alzahrani, Alhanouf Alalyani, Raghad Almutairi, Hussam Hijazi, Farag Shuweihdi, Lojain Abdulaal, Bashair Alhummiany, Naglaa Elsayed, Awad Alzahrani, Meshal Alzahrani, Nasser M. Alzahrani, Lamyaa Aljaafari, Hanan M. Alzahrani, Omar Iskanderani

**Affiliations:** 1 Department of Radiological Sciences, Faculty of Applied Medical Sciences, King Abdulaziz University, Jeddah 21589, Saudi Arabia; 2 Department of Radiology, Faculty of Medicine, King Abdulaziz University, Jeddah 21589, Saudi Arabia; 3 Faculty of Health and Life Sciences, University of Leeds, Leeds LS2 9JT, UK; 4 Libyan Authority for Scientific Research, Tripoli, Libya; 5 Department of Diagnostic Radiology, College of Applied Medical Sciences, Taibah University, Madinah 42353, Saudi Arabia; 6 Diagnostic Radiology Department, Faculty of Medicine, Cairo University, Cairo 11956, Egypt; 7 Department of Radiology and Medical Imaging, College of Applied Medical Sciences, King Saud Bin Abdulaziz University for Health Sciences, Al Ahsa 31982, Saudi Arabia; 8 King Abdullah International Medical Research Center, Al Ahsa 31982, Saudi Arabia

**Keywords:** rectal cancer, radiotherapy, toxicity, hemoglobin, leukocytes, body mass index

## Abstract

**Background/Objectives**: Radiotherapy is an important component of rectal cancer treatment, but treatment-related toxicity varies considerably between patients, suggesting that patient-related factors may influence susceptibility. Pretreatment hemoglobin, leukocyte count, and body mass index (BMI) are routinely available measures, yet their associations with acute and late toxicity during and after radiotherapy remain unclear. This study evaluated these associations in patients with rectal cancer. **Methods**: A retrospective analysis included 67 patients with histologically confirmed rectal cancer who underwent radiotherapy. Acute toxicity occurred during radiotherapy or within 3 months after treatment completion, and late toxicity occurred thereafter. Toxicity was graded using the Common Terminology Criteria for Adverse Events (CTCAE) and dichotomized as Grades 0–1 or Grade ≥ 2. Pretreatment hemoglobin, total leukocyte count, and BMI were analyzed as continuous variables. Logistic regression examined associations with acute and late toxicity, with sequential adjustment for age, sex, and radiotherapy course. **Results**: Grade ≥ 2 acute toxicity occurred in 17 patients (25.4%), and Grade ≥ 2 late toxicity in 27 (40.3%). Higher pretreatment leukocyte count was associated with greater odds of acute toxicity in the fully adjusted model (OR 1.70, 95% CI 1.13–2.58; *p* = 0.012). Higher pretreatment hemoglobin was associated with lower odds of late toxicity in the fully adjusted model (OR 0.57, 95% CI 0.40–0.81; *p* = 0.002). No clear association was observed between BMI and either acute or late toxicity. **Conclusions**: Pretreatment leukocyte count was associated with acute toxicity, while pretreatment hemoglobin was associated with late toxicity. These inexpensive, routinely available measures may represent candidate markers of toxicity, although their clinical utility requires validation in larger, independent studies.

## Simple Summary

Radiotherapy is an important component of rectal cancer treatment, but treatment-related toxicity varies considerably between patients. Patient-related factors, such as pretreatment hemoglobin, leukocyte count, and body mass index, may contribute to this variability. This study investigated whether these pretreatment patient-related factors were associated with toxicity occurring during and after radiotherapy in patients with rectal cancer. Higher pretreatment leukocyte count was associated with increased odds of Grade ≥ 2 acute toxicity, whereas higher pretreatment hemoglobin was associated with reduced odds of Grade ≥ 2 late toxicity. The analyses did not provide clear evidence of an association between body mass index and either acute or late toxicity. These findings highlight pretreatment hemoglobin and leukocyte count as potential markers of toxicity, with larger independent studies needed to validate their clinical utility.

## 1. Introduction

Colorectal cancer remains a major health challenge globally, with the third highest incidence and the second highest cancer-related mortality worldwide [1]. The management of rectal cancer involves multimodal treatment strategies, including surgery, systemic therapy, and radiotherapy [2,3]. Treatment selection depends on disease stage, tumor characteristics, and individual patient factors [2,3,4].

Radiotherapy is widely used in the treatment of locally advanced rectal cancer, particularly in the neoadjuvant setting [2,3]. Despite advances in radiotherapy technology, treatment planning, and delivery, radiotherapy-related toxicity remains an important clinical concern for patients with rectal cancer [5,6]. Radiotherapy-related toxicities are generally classified according to their time of onset. Acute toxicities occur during treatment or within 3 months of radiotherapy, whereas late toxicities develop beyond this period and may emerge months or even years later [7,8]. Common acute toxicities include diarrhea, fatigue, dermatitis, and urinary symptoms [5,9], while late effects may include genitourinary dysfunction, fecal incontinence, chronic bowel dysfunction, rectal bleeding, and pelvic fractures [5,10]. Late toxicity is particularly relevant because it may persist long after treatment and adversely affect long-term function and quality of life [11,12]. Severe Grade ≥ 3 late gastrointestinal toxicity has been reported in up to 19% of patients following radiotherapy for rectal cancer, highlighting the potential long-term burden of treatment-related toxicity [13].

Previous research on radiotherapy toxicity has largely focused on treatment-related, dosimetric, and tumor-related factors [6,9,13,14,15]. Although these factors are important determinants of toxicity, they may not fully explain the considerable variation observed between individual patients. Patients with apparently similar tumor characteristics and treatment parameters can experience markedly different degrees of toxicity [16]. This variability suggests that baseline patient-related factors may also influence susceptibility to radiotherapy-related toxicity. Among these baseline factors, pretreatment hemoglobin, leukocyte count, and BMI have been investigated in relation to clinical outcomes in rectal cancer.

Pretreatment hemoglobin has been investigated primarily as a prognostic marker in patients with rectal cancer undergoing neoadjuvant chemoradiotherapy. Lower pretreatment hemoglobin levels and anemia have been associated with poorer overall survival [17,18,19] and with poorer pathological response and local control [19,20,21]. Pelvic chemoradiotherapy can also produce substantial hematological changes, including reductions in hemoglobin and circulating leukocytes [22]. Similarly, pretreatment leukocyte count has been investigated as a prognostic marker in rectal cancer, with higher levels associated with poorer survival outcomes [17]. Body mass index (BMI) and body-composition characteristics have also been investigated in relation to treatment tolerance. Higher BMI has been associated with radiotherapy interruption in patients with locally advanced rectal cancer [23], while BMI has also been incorporated into a predictive model for severe acute toxicity during neoadjuvant chemoradiotherapy [6].

Previous studies in cervical and prostate cancers have also examined hematological and inflammatory markers in relation to normal-tissue toxicity. In cervical cancer, hemoglobin levels during treatment have been associated with acute toxicity [24], while studies in prostate cancer have explored early hematological changes [25] and routine inflammatory markers in relation to urinary and bowel toxicity [26].

However, the associations of these pretreatment measures with acute and late toxicity during and after radiotherapy are not well established, particularly for late toxicity. As these measures are routinely available before treatment, clarifying their associations with toxicity could provide additional information relevant to toxicity monitoring without requiring specialized testing. Therefore, the primary aim of this study was to evaluate the associations of pretreatment hemoglobin, total leukocyte count, and BMI with acute and late toxicity during and after radiotherapy in patients with rectal cancer.

## 2. Materials and Methods

### 2.1. Study Design and Patients

A retrospective analysis was conducted on 67 patients with histologically confirmed rectal cancer who underwent radiotherapy at King Abdulaziz University Hospital, Jeddah, Saudi Arabia, between 2021 and 2025. A total of 91 consecutive patients were screened for eligibility, of whom 24 were excluded: 22 received palliative-intent radiotherapy, as documented in their radiotherapy consent forms, and two had missing toxicity outcome data. The remaining 67 patients were included in the final analysis (Supplementary Figure S1). Metastatic disease (M1) was not an exclusion criterion. The 11 patients with documented M1 received pelvic radiotherapy as part of a multidisciplinary treatment strategy aimed at locoregional control rather than solely for symptom palliation. Clinical follow-up data were collected up to 31 July 2026.

Patients received either long-course radiotherapy (45–50.4 Gy in 25–28 fractions) or short-course radiotherapy (25 Gy in 5 fractions), predominantly delivered using volumetric modulated arc therapy (VMAT). Clinical, laboratory, treatment, and toxicity data were obtained from electronic medical records.

BMI was calculated as weight in kilograms divided by height in meters squared (kg/m^2^). Pretreatment hemoglobin and total leukocyte count were obtained from the closest laboratory measurements within 14 days before the initiation of radiotherapy.

Toxicity occurring during and after radiotherapy was graded according to the National Cancer Institute’s Common Terminology Criteria for Adverse Events (CTCAE), version 5.0, based on documented symptoms, including gastrointestinal, rectal, dermatologic, and genitourinary adverse events. Acute toxicity data were obtained from the institutional radiotherapy information system, while late toxicity data were obtained from physician follow-up records. Toxicity grading was performed by two radiation oncologists who were blinded to patients’ pretreatment laboratory results. For each patient, the overall toxicity grade for each period was defined as the highest CTCAE grade recorded among all documented adverse events during that period. Acute toxicity was defined as toxicity occurring during radiotherapy or within 3 months after treatment completion, whereas late toxicity was defined as toxicity occurring more than 3 months after treatment completion. Grade 0 classification was based on clinical follow-up notes explicitly documenting that patients were “doing well” or had “no complaints.” For the patients who died during follow-up, toxicity was assessed using the last available clinical evaluation before death. For statistical analysis, toxicity grades were dichotomized as none-to-mild (Grades 0–1) and moderate-to-severe (Grade ≥ 2).

### 2.2. Statistical Analysis

Statistical analyses were conducted using IBM SPSS version 28.0 (IBM Corp., Armonk, NY, USA). Additional sensitivity analyses, including Firth penalized logistic regression, restricted cubic splines, Box–Tidwell assessments, and leave-one-out analyses, were performed using R software (version 4.6.1; R Foundation for Statistical Computing, Vienna, Austria). Continuous variables were reported as mean with standard deviation (SD) or median with interquartile range (IQR), depending on their distribution. Categorical variables were presented as frequencies and percentages.

Acute and late toxicities were analyzed as binary outcomes, comparing CTCAE Grade ≥ 2 with Grades 0–1. Pretreatment hemoglobin, total leukocyte count, and BMI were analyzed as continuous variables.

Binary logistic regression was used to evaluate the association of each pretreatment measure with acute and late toxicity. Each pretreatment measure was analyzed separately for each toxicity outcome. Odds ratios (ORs) with 95% CIs were used to estimate the change in odds of Grade ≥ 2 toxicity associated with a 1 g/dL increase in hemoglobin, a 1 × 10^9^/L increase in leukocyte count, or a 1 kg/m^2^ increase in BMI. Three sequential models were used for each pretreatment measure. Model 1 included the pretreatment measure alone, Model 2 adjusted for age and sex, and Model 3 (the primary adjusted model) additionally adjusted for radiotherapy course (long-course vs. short-course). Age, sex, and radiotherapy course were prespecified as adjustment variables based on their clinical relevance. Separate models were used to limit model complexity given the sample size.

BMI was missing for 6 of 67 patients (9.0%), hemoglobin for 8 (11.9%), and leukocyte count for 9 (13.4%). Missing data were handled using multiple imputation with predictive mean matching (PMM). Five imputed datasets were generated using 10 iterations. The imputation procedure was conducted under a missing-at-random (MAR) assumption. The imputation model included pretreatment BMI, hemoglobin, leukocyte count, age, sex, radiotherapy course, and the acute and late Grade ≥ 2 toxicity outcomes. Each prespecified logistic regression model was fitted separately within the five imputed datasets. Regression coefficients and their associated variances were subsequently pooled using Rubin’s rules to obtain pooled odds ratios (ORs), 95% confidence intervals (CIs), and *p* values. Because the MAR assumption cannot be verified empirically, complete-case analyses using the same regression models were also performed as sensitivity analyses.

The distributions of the continuous pretreatment measures were examined for extreme values. For acute toxicity, linearity in the logit was assessed using the Box–Tidwell approach in complete-case analyses. Additional post hoc complete-case sensitivity analyses explored potential nonlinearity in the two associations of interest using restricted cubic splines with three knots placed at the 10th, 50th, and 90th percentiles of the observed biomarker distributions. Leave-one-out sensitivity analyses were performed to assess the influence of individual observations on the principal associations. Given the limited number of Grade ≥ 2 toxicity events, more complex nonlinear models were not incorporated into the primary analyses to limit overfitting.

Given the limited number of Grade ≥ 2 acute toxicity events, Firth penalized logistic regression was additionally performed as a sensitivity analysis to reduce potential small-sample bias. The penalized models used the same covariates as Model 3 (age, sex, and radiotherapy course), with each pretreatment measure evaluated separately.

Additional analyses examined differences between radiotherapy courses. Pretreatment leukocyte counts were compared between short- and long-course radiotherapy using the Mann–Whitney U test, while the proportions of patients experiencing Grade ≥ 2 acute toxicity were compared using Fisher’s exact test. The interval from radiotherapy initiation to clinically documented Grade ≥ 2 acute toxicity was calculated and summarized separately by radiotherapy course.

The six exposure–outcome associations were exploratory, with no prespecified primary hypothesis. Model 3 was the main adjusted model for interpretation. No correction for multiple comparisons was applied. These associations were interpreted primarily based on effect estimates and 95% CIs, with *p* values reported descriptively. All statistical tests were two-sided. For the additional comparisons between radiotherapy courses, *p* values were assessed using a nominal threshold of 0.05. During the preparation of this manuscript, the authors used ChatGPT (GPT-6, OpenAI) for language editing, proofreading, table formatting, and figure visualization to improve clarity and presentation. The authors reviewed and verified all AI-assisted outputs and take full responsibility for the final content.

## 3. Results

### 3.1. Patient and Treatment Characteristics

Baseline patient, tumor, and treatment characteristics are summarized in Table 1. The mean age was 61.8 years (SD = 12.5) and 58.2% of patients were male. T3 and N2 were the most common T and N stages (56.4% and 65.5%, respectively). The mean BMI was 26.7 kg/m^2^ (SD = 6.1), while the median pretreatment hemoglobin and leukocyte count were 10.5 g/dL (IQR, 9.7–12.9) and 6.22 × 10^9^/L (IQR, 5.3–7.4), respectively. Long-course radiotherapy was delivered to 67.2% of patients, and VMAT was the predominant radiotherapy technique (82.1%).

### 3.2. Associations of Pretreatment Measures with Acute Toxicity

**Figure 1:**
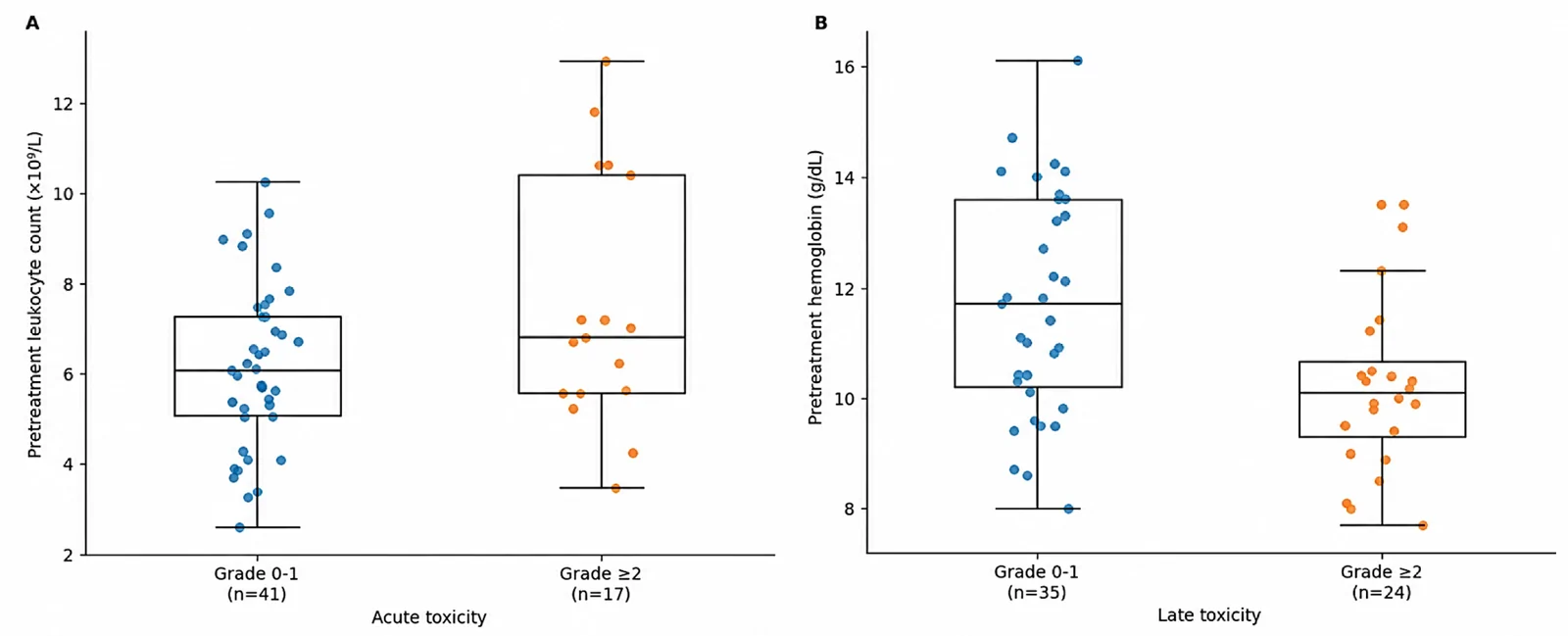
Boxplots showing distribution of pretreatment measures according to acute and late toxicity severity based on observed data. (**A**) Pretreatment leukocytes count according to acute toxicity (Grade 0–1, *n* = 41; Grade ≥ 2, *n* = 17). (**B**) Pretreatment hemoglobin according to late toxicity (Grade 0–1, *n* = 35; Grade ≥ 2, *n* = 24). The horizontal line within each box represents the median, and the lower and upper boundaries represent the first and third quartiles (25th and 75th percentiles), respectively. Observations beyond the whiskers are identified as outliers. Individual patient values are shown as dots.

Grade ≥ 2 acute toxicity occurred in 17 of 67 patients (25.4%). Higher pretreatment leukocyte count was associated with greater odds of Grade ≥ 2 acute toxicity (Table 2; Figure 1A). For the unadjusted model, each 1 × 10^9^/L increase in leukocyte count was associated with 32% higher odds of Grade ≥ 2 acute toxicity (OR, 1.315; 95% CI, 1.001–1.727; *p* = 0.049). The association was also observed after adjustment for age and sex (OR, 1.363; 95% CI, 1.005–1.852; *p* = 0.047) and after additional adjustment for radiotherapy course (OR, 1.704; 95% CI, 1.127–2.577; *p* = 0.012), corresponding to 70% higher odds of Grade ≥ 2 acute toxicity in the fully adjusted model.

The fully adjusted estimates for pretreatment hemoglobin and BMI did not provide clear evidence of associations with acute toxicity (*p* = 0.639 and *p* = 0.289, respectively) (Table 2). The distributions of pretreatment leukocyte count, BMI, and hemoglobin according to acute toxicity severity are presented in Figure 1A and Supplementary Figure S2A,B, respectively.

Grade ≥ 2 acute toxicity was observed in 2 of 22 patients (9.1%) receiving short-course radiotherapy and 15 of 45 patients (33.3%) receiving long-course radiotherapy (Fisher’s exact *p* = 0.039). Median pretreatment leukocyte count was 6.42 × 10^9^/L (IQR 5.64–9.07) in the short-course group and 6.05 × 10^9^/L (IQR 5.23–7.26) in the long-course group (*p* = 0.370).

The median interval from radiotherapy initiation to the first documented Grade ≥ 2 acute toxicity was 2.5 days (range: 2–3) for short-course radiotherapy and 34 days (IQR: 25–38) for long-course radiotherapy. Despite the different treatment durations, acute toxicity events were documented close to radiotherapy completion in both groups.

Further details on toxicity outcomes and pretreatment hemoglobin, leukocyte count, and BMI by radiotherapy course are provided in Supplementary Table S1. The distribution of acute toxicity by organ system, adverse event, and CTCAE grade is presented in Table 3.

### 3.3. Associations of Pretreatment Measures with Late Toxicity

All 67 patients had documented follow-up extending beyond three months after radiotherapy completion, with a median follow-up duration of 252 days (range, 94–1098 days). Three deaths occurred during the follow-up period, and no patients were lost to follow-up before completing the minimum required follow-up period of three months after radiotherapy. Grade ≥ 2 late toxicity occurred in 27 patients (40.3%).

Higher pretreatment hemoglobin was associated with lower odds of Grade ≥ 2 late toxicity (Table 2; Figure 1B). In the unadjusted model, each 1 g/dL increase in hemoglobin was associated with 36% lower odds of Grade ≥ 2 late toxicity (OR, 0.639; 95% CI, 0.467–0.874; *p* = 0.005). The association was also observed after adjustment for age and sex (OR, 0.607; 95% CI, 0.439–0.841; *p* = 0.003) and additional adjustment for radiotherapy course (OR, 0.571; 95% CI, 0.402–0.810; *p* = 0.002), corresponding to 43% lower odds of Grade ≥ 2 late toxicity in the fully adjusted model.

Leukocyte count showed similar effect estimates across the sequential models, with ORs of 1.350 in Models 1 and 1.351 in Model 2 and 1.332 in the fully adjusted Model 3 (95% CI, 0.997–1.780; *p* = 0.053). The confidence interval for the fully adjusted estimate included 1, indicating uncertainty regarding the association. No clear association was observed between BMI and late toxicity in any model (Table 2). The distributions of pretreatment BMI and leukocyte count according to late toxicity severity are presented in Supplementary Figure S2C,D. The distribution of late toxicity by organ system, adverse event, and CTCAE grade is summarized in Table 3.

### 3.4. Sensitivity Analysis

All conventional logistic regression models converged successfully, with no evidence of complete or quasi-complete separation. Complete-case sensitivity analyses yielded findings that were generally consistent with the multiple-imputation analyses. In the fully adjusted model, pretreatment leukocyte count remained associated with acute Grade ≥ 2 toxicity (complete-case OR 1.680, 95% CI 1.120–2.530; *p* = 0.012), compared with an OR of 1.704 (95% CI 1.127–2.577; *p* = 0.012) following multiple imputation. Similarly, higher pretreatment hemoglobin remained associated with lower odds of late Grade ≥ 2 toxicity (complete-case OR 0.590, 95% CI 0.410–0.850; *p* = 0.004), compared with an OR of 0.571 (95% CI 0.402–0.810; *p* = 0.002) following multiple imputation.

Given the limited number of Grade ≥ 2 acute toxicity events, Firth penalized logistic regression was performed as a sensitivity analysis using the same covariates as Model 3. The association between pretreatment leukocyte count and Grade ≥ 2 acute toxicity was also observed in the Firth penalized regression analysis (OR 1.58, 95% CI 1.09–2.28; *p* = 0.015), while the estimates for hemoglobin and BMI provided less evidence of an association with acute toxicity.

In complete-case analyses, Box–Tidwell tests did not provide clear evidence of departures from linearity in the logit for hemoglobin (*p* = 0.633), leukocyte count (*p* = 0.133), or BMI (*p* = 0.197) in relation to acute toxicity. Additional exploratory restricted cubic spline analyses of the fully adjusted models did not provide clear evidence of nonlinearity for leukocyte count in relation to acute Grade ≥ 2 toxicity (*p* = 0.123; *n* = 58) or hemoglobin in relation to late Grade ≥ 2 toxicity (*p* = 0.662; *n* = 59). Leave-one-out analyses of these two associations showed consistent directions of effect, with adjusted odds ratios ranging from 1.58 to 2.03 for leukocyte count and from 0.53 to 0.61 for hemoglobin, respectively.

## 4. Discussion

The aim of this study was to evaluate the associations of pretreatment hemoglobin, leukocyte count, and BMI with acute and late toxicity during and after radiotherapy in patients with rectal cancer. The results showed that higher pretreatment leukocyte count was associated with greater odds of Grade ≥ 2 acute toxicity, while higher pretreatment hemoglobin was associated with lower odds of Grade ≥ 2 late toxicity. BMI was not associated with either acute or late toxicity. These findings should be interpreted in the context of the exploratory nature of the study.

Previous studies in rectal cancer have focused largely on circulating leukocytes in relation to hematological changes during treatment or oncological outcomes [17,22], with less attention to clinical radiotherapy-related toxicity. Yang et al. reported changes in circulating blood cells during pelvic chemoradiotherapy, with white blood cell counts reaching their lowest level during the second week of radiotherapy and showing associations with pelvic bone-marrow dose [22]. Our findings add to this by showing that pretreatment leukocyte count was associated with Grade ≥ 2 acute toxicity. Each 1 × 10^9^/L increase was associated with 70% higher odds in the fully adjusted model (OR 1.70, 95% CI 1.13–2.58). The interpretation of this association also requires consideration of radiotherapy course, which was associated with acute toxicity in our cohort. The increase in the leukocyte odds ratio from 1.36 to 1.70 after adjustment for radiotherapy course may be partly explained by differences in acute Grade ≥ 2 toxicity between the short- and long-course groups, despite similar pretreatment leukocyte distributions. However, the small number of acute events, particularly in the short-course group, should be considered when interpreting this change. Beyond these statistical considerations, the biological basis of this association remains uncertain. A higher pretreatment leukocyte count may reflect differences in baseline inflammatory or immune status. Inflammatory signaling, oxidative stress, and immune-cell responses contribute to radiation-induced normal-tissue injury [27,28] and may help explain the observed association.

Previous studies in rectal cancer have examined hemoglobin mainly in relation to tumor response and oncological outcomes rather than normal-tissue toxicity [17,18,20]. In our study, each 1 g/dL increase in pretreatment hemoglobin was associated with 43% lower odds of Grade ≥ 2 late toxicity after adjustment for age, sex, and radiotherapy course. This finding suggests that pretreatment hemoglobin may also be relevant to long-term treatment effects. Late radiation injury involves vascular injury, chronic inflammation, tissue hypoxia, and fibrosis [7,27,29], with experimental studies of irradiated rectal tissue also reporting hypoxia-related changes alongside fibrosis [30]. These processes provide a possible biological link between oxygen delivery and late tissue response. However, hemoglobin is influenced by nutritional status, inflammation, bleeding, and cancer-related anemia [31]. The relatively low median pretreatment hemoglobin level (10.5 g/dL) in our cohort may be related to tumor-related bleeding, inflammation, or disease burden, although the causes of anemia were not documented. Other factors, such as medication use, blood transfusions, and active infections, may also have affected pretreatment blood values and contributed to residual confounding. Unlike hemoglobin and leukocyte count,, no clear association was observed between BMI and either acute or late toxicity. Previous studies, however, suggest that body composition may still be relevant to treatment tolerance. Chiloiro et al. studied 628 patients with locally advanced rectal cancer and found that higher BMI and lower skeletal muscle index were associated with radiotherapy interruption [23]. BMI alone may not fully reflect body composition because it does not distinguish between skeletal muscle and adipose tissue [32]. CT-based body-composition measures may therefore provide a better understanding of how body composition relates to radiotherapy-related toxicity [33]. Recent evidence further suggests that changes in skeletal muscle measures during chemoradiotherapy may be associated with treatment-related adverse events in patients with rectal cancer [34].

The rate of severe late toxicity also warrants consideration. Grade 3 late toxicity was documented in 20 patients (29.9%), including gastrointestinal and non-gastrointestinal events. Grade 3 gastrointestinal toxicity was recorded in 17 patients (25.4%), compared with rates of up to 19% reported in a previous systematic review [13]. Differences in toxicity assessment and treatment characteristics may contribute to this variation. Surgery may also have contributed to late symptoms, particularly as 24 of the 32 patients who underwent surgery had their procedure after radiotherapy. Therefore, these events cannot be attributed solely to radiotherapy.

Sensitivity analyses provided further support for the consistency of the main findings. The exploratory analyses did not provide clear evidence of nonlinearity in the association between pretreatment leukocyte count and acute toxicity, although a nonlinear relationship cannot be excluded given the sample size. Leave-one-out analyses showed that excluding individual patients did not substantially alter the main associations. Furthermore, the association between pretreatment leukocyte count and acute Grade ≥ 2 toxicity was also observed following Firth penalization, although the estimated OR was slightly lower. These findings suggest that the observed association was not driven by individual observations or solely by the estimation method. Nevertheless, the precision of the estimated effect remains limited by the relatively small number of acute toxicity events.

This study evaluated three pretreatment measures with both acute and late toxicity during and after radiotherapy, analyzed them as continuous variables, and used prespecified adjustment for age, sex, and radiotherapy course. However, the retrospective study design, single-center setting, and relatively small sample size may limit the generalizability of the findings. The limited number of Grade ≥ 2 toxicity events, particularly acute events, restricted the number of variables included in the multivariable models, while retrospective toxicity assessment may have been affected by differences in symptom documentation. Although all patients had documented follow-up beyond three months after radiotherapy completion, differences in follow-up duration should be considered when interpreting the observed late-toxicity outcomes.

Another limitation was the incomplete availability of clinical and treatment-related information. Although this study focused on pretreatment measures to complement previous research on treatment-related and dosimetric factors, these factors may have influenced the observed associations. Chemotherapy exposure data were available for 66 patients, although detailed treatment information was incomplete, while surgical information was available for 32 patients. The timing of blood sampling relative to systemic therapy could not be established for all patients. Baseline clinical characteristics were not consistently documented, and patient-level organ-at-risk dosimetric parameters were not collected. Most patients received VMAT (82.1%), limiting comparisons between radiotherapy techniques. Given the small cohort and limited number of toxicity events, including additional variables in the multivariable models could have increased the risk of overfitting. Consequently, residual confounding cannot be excluded, and adverse events could not always be attributed specifically to radiotherapy rather than other treatment components, particularly for late toxicity.

Multiple imputation was used to address missing pretreatment measurements, although the missing-at-random assumption could not be verified. Importantly, complete-case analyses yielded effect estimates comparable to those obtained following multiple imputation, providing some reassurance regarding the consistency of the main findings. Nevertheless, given the multiple exposure–outcome comparisons without formal adjustment for multiplicity, the results should be interpreted as exploratory and hypothesis-generating.

Future studies could incorporate more detailed treatment and dosimetric characteristics, differential leukocyte counts, inflammatory markers, longitudinal hemoglobin measurements, and CT-derived body-composition measures to clarify these associations and their potential role in toxicity monitoring and prevention.

## 5. Conclusions

This study identified associations between pretreatment measures and acute and late toxicity during and after radiotherapy in patients with rectal cancer. Higher pretreatment leukocyte count was associated with increased odds of Grade ≥ 2 acute toxicity, whereas higher pretreatment hemoglobin was associated with reduced odds of Grade ≥ 2 late toxicity. These findings suggest that pretreatment leukocyte count and hemoglobin may serve as candidate markers for toxicity assessment. Further studies in larger, independent cohorts are needed to validate these associations and evaluate their potential role in toxicity monitoring and supportive care for patients with rectal cancer.

## Figures and Tables

**Table 1:** Baseline patient, tumor and treatment characteristics.

Characteristic	Value
**Age, years**	61.8 ± 12.5
**Sex, *n*** (**%**)	
Male	39 (58.2)
Female	28 (41.8)
**BMI, kg/m^2^**	26.7 ± 6.1
**Pretreatment hemoglobin, g/dL**	10.5 (9.7–12.9)
**Pretreatment leukocyte count, ×10^9^/L**	6.22 (5.3–7.4)
**Clinical T stage, *n*** (**%**)	
T1	1 (1.8)
T2	5 (9.1)
T3	31 (56.4)
T4	18 (32.7)
**Clinical N stage, *n*** (**%**)	
N0	10 (18.2)
N1	9 (16.4)
N2	36 (65.5)
**Clinical M stage, *n*** (**%**)	
M0	44 (80.0)
M1	11 (20.0)
**Radiotherapy course, *n*** (**%**)	
Long-course	45 (67.2)
Short-course	22 (32.8)
**Radiotherapy technique, *n*** (**%**)	
VMAT	55 (82.1)
IMRT	4 (6.0)
3D-CRT	8 (11.9)
**Chemotherapy, *n*** (**%**)	
Yes	53 (80.3)
No	13 (19.7)
**Surgery**	
**Surgery timing, *n*** (**%**)	
Pre-radiotherapy	8 (25.0)
Post-radiotherapy	24 (75.0)
**Surgical procedure type, *n*** (**%**)	
Low anterior resection (LAR)	21 (65.6)
Abdominoperineal resection (APR)	6 (18.8)
Other surgical procedures	5 (15.6)
**Stoma status, *n*** (**%**)	
Yes	9 (31.0)
No	20 (69.0)

Data are presented as mean ± SD, median (IQR), or *n* (%). Available sample sizes were BMI, *n* = 61; hemoglobin, *n* = 59; leukocyte count, *n* = 58; clinical TNM stage, *n* = 55; chemotherapy, *n* = 66; surgery timing, *n* = 32; surgical procedure type, *n* = 32; and stoma status, *n* = 29. All other variables were available for all 67 patients. Missing values were excluded from percentage calculations. Other surgical procedures included proctosigmoidectomy, transanal excision, and submucosal excision of a rectal lesion. BMI, body mass index; IQR, interquartile range; SD, standard deviation; VMAT, volumetric-modulated arc therapy; IMRT, intensity-modulated radiotherapy; 3D-CRT, three-dimensional conformal radiotherapy; LAR, low anterior resection; APR, abdominoperineal resection.

**Table 2:** Associations of pretreatment measures with acute and late toxicity during and after radiotherapy in patients with rectal cancer.

Outcome/Pretreatment Measure	Grade 0–1 *n* (%)	Grade ≥ 2 *n* (%)	Model 1 OR (95% CI)	*p* Value	Model 2 OR (95% CI)	*p* Value	Model 3 OR (95% CI)	*p* Value
**Acute toxicity**	50 (74.6)	17 (25.4)						
Hemoglobin, per 1 g/dL			0.989 (0.751–1.302)	0.937	0.883 (0.652–1.194)	0.417	0.927 (0.673–1.275)	0.639
Leukocyte count, per 1 × 10^9^/L			1.315 (1.001–1.727)	0.049	1.363 (1.005–1.852)	0.047	1.704 (1.127–2.577)	0.012
BMI, per 1 kg/m^2^			0.921 (0.831–1.019)	0.111	0.943 (0.844–1.052)	0.293	0.943 (0.847–1.051)	0.289
**Late toxicity**	40 (59.7)	27 (40.3)						
Hemoglobin, per 1 g/dL			0.639 (0.467–0.874)	0.005	0.607 (0.439–0.841)	0.003	0.571 (0.402–0.810)	0.002
Leukocyte count, per 1 ×10^9^/L			1.350 (1.014–1.796)	0.040	1.351 (1.015–1.797)	0.040	1.332 (0.997–1.780)	0.053
BMI, per 1 kg/m^2^			1.061 (0.975–1.154)	0.168	1.072 (0.979–1.173)	0.134	1.074 (0.979–1.179)	0.129

Model 1: unadjusted; Model 2: adjusted for age and sex; Model 3: adjusted for age, sex, and radiotherapy course (long-course vs. short-course). All models included 67 patients and were fitted using multiply imputed datasets.

**Table 3:** Maximum CTCAE v5.0 toxicity grade per patient and representative adverse events during acute and late assessment (*n* = 67).

Organ System/Adverse Event	Grade 1 *n* (%)	Grade 2 *n* (%)	Grade 3 *n* (%)	Total *n* (%)
**A. Acute toxicity—during radiotherapy or within 3 months after completion**				
**Gastrointestinal**	**14** (**20.9**)	**13** (**19.4**)	**1** (**1.5**)	**28** (**41.8**)
Abdominal pain	5 (7.5)	2 (3.0)	0 (0.0)	7 (10.4)
Bowel obstruction	0 (0.0)	1 (1.5)	0 (0.0)	1 (1.5)
Constipation	0 (0.0)	1 (1.5)	0 (0.0)	1 (1.5)
Diarrhea	6 (9.0)	1 (1.5)	0 (0.0)	7 (10.4)
Hemorrhoids	1 (1.5)	0 (0.0)	0 (0.0)	1 (1.5)
Nausea	1 (1.5)	2 (3.0)	0 (0.0)	3 (4.5)
Vomiting	1 (1.5)	0 (0.0)	0 (0.0)	1 (1.5)
Proctitis	0 (0.0)	1 (1.5)	0 (0.0)	1 (1.5)
Rectal pain	0 (0.0)	1 (1.5)	1 (1.5)	2 (3.0)
Rectal hemorrhage	0 (0.0)	3 (4.5)	0 (0.0)	3 (4.5)
Anal hemorrhage	0 (0.0)	1 (1.5)	0 (0.0)	1 (1.5)
**Renal and urinary**	**5** (**7.5**)	**2** (**3.0**)	**0** (**0.0**)	**7** (**10.4**)
Dysuria	3 (4.5)	2 (3.0)	0 (0.0)	5 (7.5)
Hematuria	1 (1.5)	0 (0.0)	0 (0.0)	1 (1.5)
Nocturia	1 (1.5)	0 (0.0)	0 (0.0)	1 (1.5)
**Skin and subcutaneous tissue**	**1** (**1.5**)	**0** (**0.0**)	**0** (**0.0**)	**1** (**1.5**)
Dermatitis radiation	1 (1.5)	0 (0.0)	0 (0.0)	1 (1.5)
**Blood and lymphatic**	**0** (**0.0**)	**0** (**0.0**)	**1** (**1.5**)	**1** (**1.5**)
Anemia	0 (0.0)	0 (0.0)	1 (1.5)	1 (1.5)
**Nervous system**	**1** (**1.5**)	**0** (**0.0**)	**0** (**0.0**)	**1** (**1.5**)
Dizziness	1 (1.5)	0 (0.0)	0 (0.0)	1 (1.5)
**Overall toxicity** (**Grades 1–3**)	**21** (**31.3**)	**15** (**22.4**)	**2** (**3.0**)	**38** (**56.7**)
*Grade 0 (no recorded toxicity): 29 (43.3%). Total: 67 (100%).*				
**B. Late toxicity—more than 3 months after radiotherapy completion**				
**Gastrointestinal**	**4** (**6.0**)	**3** (**4.5**)	**17** (**25.4**)	**24** (**35.8**)
Abdominal pain	2 (3.0)	1 (1.5)	3 (4.5)	6 (9.0)
Anal discharge	1 (1.5)	0 (0.0)	0 (0.0)	1 (1.5)
Bowel obstruction	0 (0.0)	0 (0.0)	1 (1.5)	1 (1.5)
Diarrhea	0 (0.0)	0 (0.0)	1 (1.5)	1 (1.5)
Hemorrhoids	0 (0.0)	0 (0.0)	1 (1.5)	1 (1.5)
Nausea	0 (0.0)	0 (0.0)	1 (1.5)	1 (1.5)
Vomiting	1 (1.5)	1 (1.5)	0 (0.0)	2 (3.0)
Rectal pain	0 (0.0)	1 (1.5)	8 (11.9)	9 (13.4)
Rectal/anal bleeding	0 (0.0)	0 (0.0)	2 (3.0)	2 (3.0)
**Renal and urinary**	**1** (**1.5**)	**3** (**4.5**)	**0** (**0.0**)	**4** (**6.0**)
Dysuria	1 (1.5)	1 (1.5)	0 (0.0)	2 (3.0)
Hematuria	0 (0.0)	2 (3.0)	0 (0.0)	2 (3.0)
**General disorders and administration site conditions**	**0** (**0.0**)	**1** (**1.5**)	**0** (**0.0**)	**1** (**1.5**)
Localized edema (perianal)	0 (0.0)	1 (1.5)	0 (0.0)	1 (1.5)
**Investigations**	**1** (**1.5**)	**0** (**0.0**)	**0** (**0.0**)	**1** (**1.5**)
Weight loss	1 (1.5)	0 (0.0)	0 (0.0)	1 (1.5)
**Blood and lymphatic**	**0** (**0.0**)	**0** (**0.0**)	**1** (**1.5**)	**1** (**1.5**)
Anemia	0 (0.0)	0 (0.0)	1 (1.5)	1 (1.5)
**Reproductive system**	**0** (**0.0**)	**0** (**0.0**)	**2** (**3.0**)	**2** (**3.0**)
Erectile dysfunction	0 (0.0)	0 (0.0)	2 (3.0)	2 (3.0)
**Overall toxicity** (**Grades 1–3**)	**6** (**9.0**)	**7** (**10.4**)	**20** (**29.9**)	**33** (**49.3**)
*Grade 0 (no recorded toxicity): 34 (50.7%). Total: 67 (100%).*				

Notes. Data are presented as n (%) of the total cohort (*n* = 67). Adverse events were grouped using standardized terminology without altering the original toxicity grades. No Grade 4 or 5 toxicities were reported.

## Data Availability

The data presented in this study are available from the corresponding author upon request.

## Supplementary Materials

The following supporting information can be downloaded at: https://www.mdpi.com/article/10.3390/cancers18213475/s1, Figure S1: Patient screening and inclusion flow diagram; Figure S2: Boxplots showing the distribution of pretreatment measures according to toxicity severity; Table S1: Comparison of toxicity outcomes and pretreatment measures by radiotherapy course.

## Abbreviations

The following abbreviations are used in this manuscript:

BMI	Body mass index
CTCAE	Common Terminology Criteria for Adverse Events
SD	Standard deviation
IQR	Interquartile range
OR	Odds ratio
CI	Confidence interval
VMAT	Volumetric-modulated arc therapy
IMRT	Intensity-modulated radiotherapy
3D-CRT	Three-dimensional conformal radiotherapy
TNM	Tumor–node–metastasis
