# Supplementary material for: Associations of Pretreatment Hemoglobin, Body Mass Index, and Leukocyte Count with Acute and Late Toxicity During and After Radiotherapy for Rectal Cancer
Source: Cancers (Basel). 2026 Oct 28;18(21):3475. doi: 10.3390/cancers18213475 (PMC13762266; doi:10.3390/cancers18213475)
Supplement: Supplementary file 1 [file cancers-18-03475-s001.zip › cancers-4621952-supplementary.pdf]

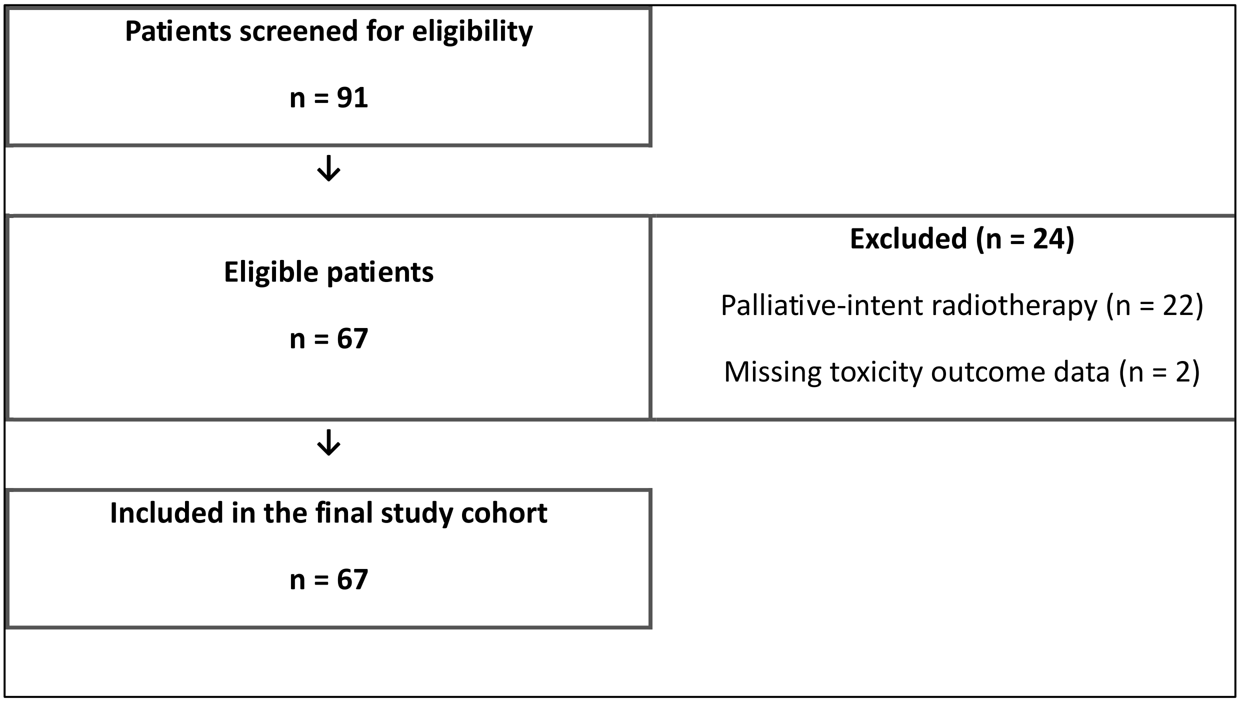

Figure S1. *Patient screening and inclusion flow diagram. Of 91 patients screened for eligibility, 24 were excluded: 22 received palliative-intent radiotherapy, as documented in radiotherapy consent forms, and two had missing toxicity outcome data. The final cohort included 67 patients.*

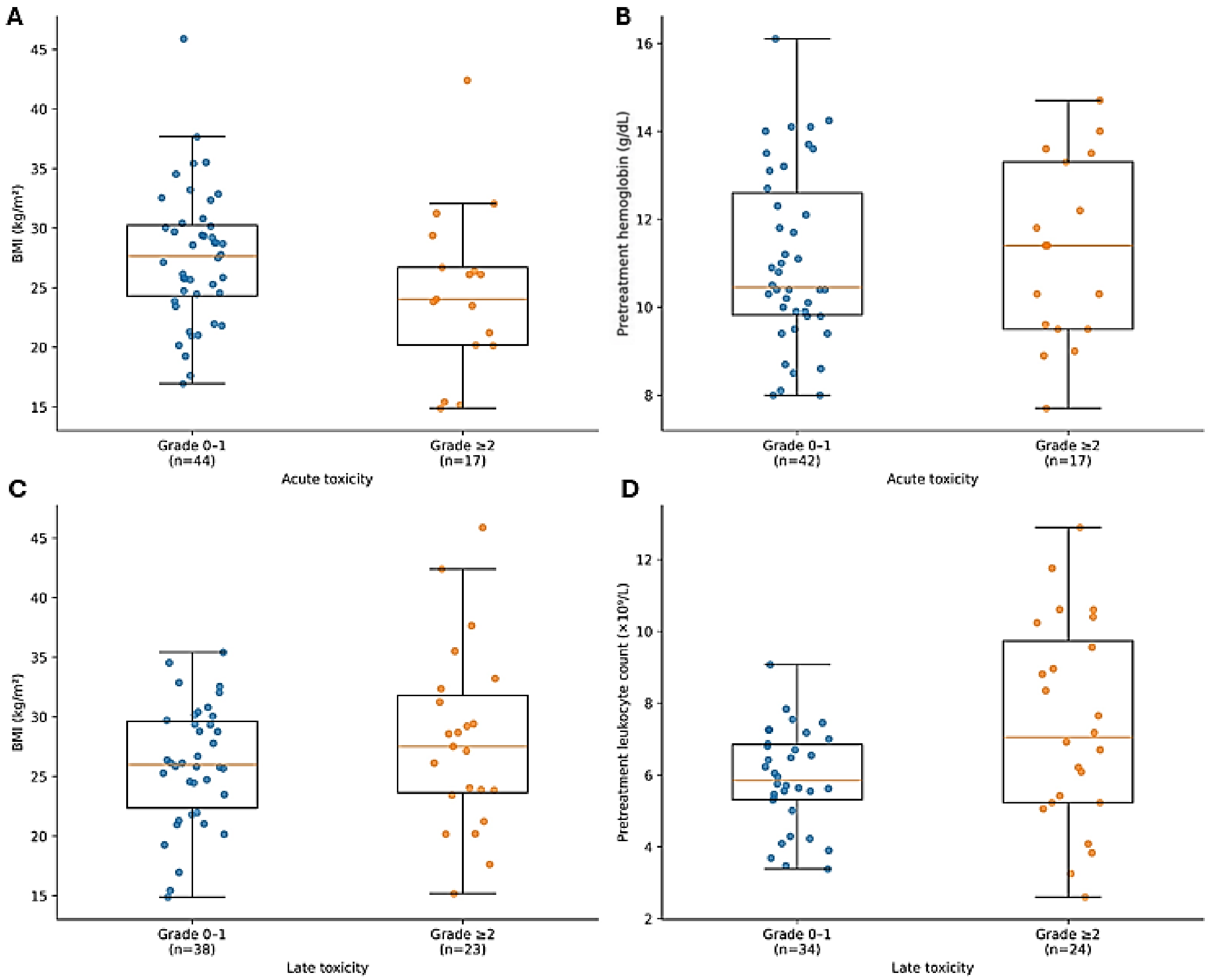

Figure S2. Boxplots showing the distribution of pretreatment measures according to toxicity severity. (A) Pretreatment body mass index (BMI) by acute toxicity grade. (B) Pretreatment hemoglobin by acute toxicity grade. (C) Pretreatment BMI by late toxicity grade. (D) Pretreatment leukocyte count by late toxicity grade. The boxes represent the interquartile range (25th–75th percentiles), with the horizontal line indicating the median. Whiskers extend to the most extreme values within 1.5 times the interquartile range (IQR). Individual patient values, including outliers, are shown as dots. The number of patients in each group (n) is indicated below the boxplots.

Supplementary Table S1: Comparison of toxicity outcomes and pretreatment measures by radiotherapy course.

Characteristic	Long-course RT	Short-course RT	p value
Patients, n	45	22	—
Acute Grade ≥2 toxicity, n (%)	15 (33.3)	2 (9.1)	0.039
Late Grade ≥2 toxicity, n (%)	16 (35.6)	11 (50.0)	0.297
Pretreatment leukocyte count, $\times 10^9$/L, median (IQR)	6.05 (5.23–7.26), n = 41	6.42 (5.64–9.07), n = 17	0.370
Pretreatment hemoglobin, g/dL, median (IQR)	10.4 (9.5–12.18), n = 42	10.8 (10.2–13.5), n = 17	0.412
BMI, kg/m^2, median (IQR)	26.37 (21.55–30.71), n = 43	25.96 (23.84–28.77), n = 18	0.831

Data are based on observed, non-imputed values. Toxicity proportions were compared using Fisher's exact test, and continuous pretreatment measures were compared using the Mann–Whitney U test. IQR, interquartile range; BMI, body mass index; RT, radiotherapy.
